# The Indianapolis Flux Experiment (INFLUX): A test-bed for developing urban greenhouse gas emission measurements

**DOI:** 10.1525/elementa.188

**Published:** 2017

**Authors:** Kenneth J. Davis, Aijun Deng, Thomas Lauvaux, Natasha L. Miles, Scott J. Richardson, Daniel P. Sarmiento, Kevin R. Gurney, R. Michael Hardesty, Timothy A. Bonin, W. Alan Brewer, Brian K. Lamb, Paul B. Shepson, Rebecca M. Harvey, Maria O. Cambaliza, Colm Sweeney, Jocelyn C. Turnbull, James Whetstone, Anna Karion

**Affiliations:** * Department of Meteorology and Atmospheric Science and the Earth and Environmental Sciences Institute, The Pennsylvania State University, University Park, Pennsylvania, US; † Department of Meteorology and Atmospheric Science, The Pennsylvania State University, University Park, Pennsylvania, US; ‡ School of Life Sciences, Arizona State University, Tempe, Arizona, US; § Cooperative Institute for Research in Environmental Sciences, University of Colorado, US; ‖ NOAA Earth Systems Research Laboratory, Boulder, Colorado, US; ¶ Laboratory for Atmospheric Research, Washington State University, Pullman, Washington, US; ** Department of Chemistry and Department of Earth, Atmospheric, and Planetary Sciences, Purdue University, West Lafayette, Indiana, US; †† Department of Chemistry, Purdue University, West Lafayette, Indiana, US; ‡‡ Department of Physics, Ateneo de Manila University, Quezon City, PH; §§ GNS Science, Rafter Radiocarbon Laboratory, Lower Hutt, NZ; ‖‖ Cooperative Institute of Research in Environmental Sciences, University of Colorado, Boulder, Colorado, US; ¶¶ National Institute of Standards and Technology, Gaithersburg, Maryland, US

**Keywords:** carbon emissions, urban emissions, carbon dioxide, methane, urban meteorology, greenhouse gas measurements

## Abstract

The objective of the Indianapolis Flux Experiment (INFLUX) is to develop, evaluate and improve methods for measuring greenhouse gas (GHG) emissions from cities. INFLUX’s scientific objectives are to quantify CO_2_ and CH_4_ emission rates at 1 km resolution with a 10% or better accuracy and precision, to determine whole-city emissions with similar skill, and to achieve high (weekly or finer) temporal resolution at both spatial resolutions. The experiment employs atmospheric GHG measurements from both towers and aircraft, atmospheric transport observations and models, and activity-based inventory products to quantify urban GHG emissions. Multiple, independent methods for estimating urban emissions are a central facet of our experimental design. INFLUX was initiated in 2010 and measurements and analyses are ongoing. To date we have quantified urban atmospheric GHG enhancements using aircraft and towers with measurements collected over multiple years, and have estimated whole-city CO_2_ and CH_4_ emissions using aircraft and tower GHG measurements, and inventory methods. Significant differences exist across methods; these differences have not yet been resolved; research to reduce uncertainties and reconcile these differences is underway. Sectorally- and spatially-resolved flux estimates, and detection of changes of fluxes over time, are also active research topics. Major challenges include developing methods for distinguishing anthropogenic from biogenic CO_2_ fluxes, improving our ability to interpret atmospheric GHG measurements close to urban GHG sources and across a broader range of atmospheric stability conditions, and quantifying uncertainties in inventory data products. INFLUX data and tools are intended to serve as an open resource and test bed for future investigations. Well-documented, public archival of data and methods is under development in support of this objective.

## 1. Introduction

### 1.1 Background

Cities concentrate population, energy usage and greenhouse gas (GHG) emissions, and a growing proportion of the global population lives in cities. Urban areas contribute a large fraction of global anthropogenic CO_2_ emissions (EIA, 2013; Seto et al., 2014). A variety of sources are contributing to increased methane (CH_4_) emissions in urban areas (Pacala et al., 2010; Bellucci et al., 2012). The increasing trend in atmospheric mole fractions of GHGs is evident from continuous monitoring (NOAA, 2016), and consistent with socio-economic data tracking global consumption of fossil fuels and the greenhouse gas inventory reports provided to the UN Framework Convention on Climate Change (UNFCCC, 2016). The UNFCCC Paris Agreement (UNFCCC, 2015) gives impetus for effective GHG mitigation actions in the coming years. Mitigating emissions from urban areas will play an important role.

Accurate and precise urban emissions measurements are needed to assess progress toward, and attainment of emission reduction targets (Nisbet and Weiss, 2010; Pacala et al., 2010; Durant et al., 2011; Ciais et al., 2014). Continuous quantification of GHG emissions at the urban scale enables rapid and independent understanding of the efficacy of mitigation measures. Such measurements, if sufficiently accurate and precise, could also supplement the data required for either market-based or regulatory-based emissions mitigation measures.

Continuous measurement of GHG emissions from cities is important for developing and improving process-based understanding of urban emissions (Kennedy et al., 2009; Marcotullio et al., 2014). Similar to ecosystems, urban systems have structure and function related to their GHG emissions, and models of urban biogeochemistry similar to current ecosystem biogeochemical models can be envisioned. Urban infrastructures last for decades to centuries; understanding urban processes could inform GHG emissions management far into the future (Creutzig et al., 2015). Understanding the emergent properties of cities and the resulting GHG emissions is needed to understand and mitigate urban emissions.

A variety of methods exist for quantifying fluxes from urban environments. Inventory-based data products are one fundamental method of understanding urban GHG emissions. Current evidence for the high density and magnitude of GHG emissions from urban areas is supported primarily by population and socio-economic data, such as those data underpinning U.S. emissions reporting (Environmental Protection Agency, 2016). In recent years a number of projects have extended inventories to spatially-gridded, time-dependent products that integrate an increasingly broad array of observations (Gurney et al., 2009, 2012; Gately et al., 2013; McDonald et al., 2014), and can span the globe by integrating emissions models and remote sensing products (Oda and Maksyutov, 2011; Asefi-Najafabady et al., 2014).

Atmospheric GHG measurements are another fundamental and independent approach for quantifying and understanding urban GHG emissions. Atmospheric measurement of GHG emissions from cities has been the focus of several recent efforts (Wunch et al., 2009; Lauvaux et al., 2013; Cambaliza et al., 2014, 2015; Bréon et al., 2015; McKain, et al., 2015; Wong et al, 2015; Lamb et al., 2016; Lauvaux et al., 2016; Feng et al. 2016). Measurements have been collected from aircraft (Cambaliza et al, 2014, 2015), towers (Bréon et al, 2015; McKain et al, 2015; Lauvaux et al, 2016), ground-based remote sensing (Wong et al., 2015) and satellite (Kort et al, 2012), and analysis methods have ranged from simple atmospheric mass-balance (Cambaliza et al, 2014, 2015) to complex mesoscale atmospheric modeling efforts merged with Bayesian inversions (Lauvaux et al, 2016). These approaches promise to provide an independent assessment of urban emissions, including quantification of changes in fluxes over time (Lauvaux et al., 2013) and identification of gaps in inventory products. Atmospheric emissions estimates have often revealed significant differences when compared with inventory assessments (e.g. Lamb et al, 2016; Lauvaux et al, 2016). These differences call for further investigation.

Atmospheric measurements that cover intermediate spatial domains often fall between these two broad categories. Point flux measurements including plume dispersion (Lavoie et al., 2015; Rella et al., 2015; Yacovitch et al., 2015), stack monitoring (Gurney et al., 2016), and enclosure-based approaches (Allen et al., 2013) can be applied to urban sources. Eddy-covariance flux measurements (Grimmond et al., 2002, Crawford and Christen, 2014), which due to the nature of atmospheric turbulence represent areas of order 1 km^2^ (Horst and Weil, 1992; Kljun et al, 2015), have also been employed in urban settings. These observations can be incorporated into whole-city or other regional GHG emissions estimates, usually as input to a “bottom-up” or inventory based flux estimates (Zavala-Araiza et al, 2015; Lamb et al., 2016).

Each approach to studying urban GHG emissions has strengths and weaknesses and, similar to the challenges inherent in measuring the carbon balance of terrestrial ecosystems (Davis, 2008), simultaneous application of multiple methods (Ogle et al, 2015; Zavala-Araiza, et al., 2015) is most likely to yield rapid progress toward understanding emissions. Quantification of urban emissions with multiple, independent methods also increases confidence in the results from each method. Ideally our measurements of urban GHG emissions, in addition to quantifying continuous emissions from the entire city, should include source attribution – the ability to identify the component of the urban system responsible for those emissions. Resolving emissions according to source (e.g. traffic, industry, electric power production, waste management, natural gas infrastructure, urban biosphere) will advance management capacity and process understanding of GHG emissions.

### 1.2 Goals and objectives of the Indianapolis Flux Experiment

The Indianapolis Flux experiment (INFLUX) seeks to advance our ability to quantify urban CO_2_ and CH_4_ emissions and to compare inventory-based emissions estimates (e.g. Gurney et al., 2012) with those derived from several atmospheric ob JPL, 2017 servation and analysis methods. INFLUX is motivated by and responds to the 2010 National Research Council (NRC) report, *Verifying Greenhouse Gas Emissions: Methods to Support International Climate Agreements* (Pacala et al., 2010), and the call for study of anthropogenic contributions to the carbon cycle raised in the U.S. Carbon Cycle Science Plan (Michalak et al., 2011). It also addresses the U.S. federal government plan to slow the rate of climate change (White House, 2014).

INFLUX brings together state-of-the-science atmospheric measurements, atmospheric modeling, and inventory-based data products to advance our ability to quantify and attribute urban CO_2_ and CH_4_ emissions. This is consistent with the NRC and subsequent recommendations for the development of new atmospheric measurement and modeling approaches along with, “Simultaneous creation of detailed bottom-up inventories of emissions for these same representative areas…” (Pacala et al., 2010).

INFLUX’s scientific objectives are to quantify CO_2_ and CH_4_ sources at 1 km^2^ resolution with a 10% or better accuracy and precision, to determine whole-city emissions with similar skill, and to achieve high (weekly or finer) temporal resolution at both spatial resolutions. The project is primarily supported by the National Institute for Standards and Technology (NIST), whose overall goal is to establish measurement and modeling methods suitable for application across the U.S. and worldwide as a means of informing mitigation efforts. Careful estimation of uncertainty, and the development of verifiable, reproducible and independent methods that would be required by GHG emissions markets or regulations are central to the research effort.

INFLUX is not intended to serve as a model to be reproduced for all cities; rather, the intent is, if possible, to oversample. That is, we aim to implement the best possible methods available in a relatively simple environment to determine the limits of current science and technology, and determine what measurements and methods are essential to achieve given performance metrics. Indianapolis was chosen as the test site because it is relatively isolated from other major CO_2_ and CH_4_ sources, is on terrain that is relatively easily simulated by meteorological models, and was the initial site for the development of an innovative urban inventory product, Hestia (Gurney et al., 2012) which provides emissions estimates at geospatial and temporal scales compatible with atmospheric observing and analysis strategies. INFLUX research will identify those components of the INFLUX observational and modeling system that are able and necessary to reach specified levels of accuracy, precision and resolution in GHG emissions estimates, and what additional methodological advances would be needed to make this feasible for multiple urban centers. INFLUX is also intended to serve as a test bed for experiments that would expand the observational and analytic methods currently available. INFLUX complements other urban experiments including, but not limited to, NIST-supported efforts in Los Angeles and the Northeast Corridor (National Institute for Standards and Technology, 2017; Jet Propulsion Laboratory, 2017).

This paper will present the methodological design of the INFLUX campaign and review some results to date (section 2), discuss the major challenges remaining in pursuit of those objectives and suggest paths forward (section 3). There are many gaseous species that contribute to the greenhouse effect. INFLUX focuses on the two primary anthropogenic greenhouse gases, CO_2_ and CH_4_. For simplicity, throughout the remainder of this article the abbreviation GHG refers to the combination of CO_2_ and CH_4_ only.

## 2. Methodological design and a brief summary of results to date

INFLUX is a cross-institutional, cooperative study that builds upon recent progress made in studying the terrestrial carbon cycle including high-accuracy, high-precision atmospheric GHG mole fraction measurements (Mays et al., 2009; Karion et al., 2013; Richardson et al., 2017; Miles et al., 2017a), regional atmospheric inversions (Lauvaux et al., 2012a, 2016), airborne measurements of urban emissions (Mays et al., 2009; Cambaliza et al., 2014, 2015; Heimburger et al., 2017), high-resolution, data-constrained atmospheric transport modeling (Deng et al., 2017; Sarmiento et al., 2017a; Gaudet et al., personal communication), applications of trace gases to distinguish anthropogenic and biogenic GHG sources (Turnbull et al., 2015; Nathan et al., 2017), and the development of high resolution, activity-based GHG emissions estimates (Gurney et al., 2012; Oda et al., 2017). Table 1 presents an overview of the key observational and modeling components that are at the core of INFLUX.

These components are intended to serve as an integrated whole to address INFLUX’s scientific objectives. Many data are archived on line and available to the public (Table 1). The following discussion (section 2.1) provides a brief summary of these elements of the investigation, followed by the approach envisioned for integration of these elements (section 2.2). Brief syntheses of results to date are summarized in each section.

### 2.1 Methodological components

#### 2.1.1 Atmospheric GHG observations

Atmospheric GHG observations are the most fundamental element in our effort to infer urban GHG emissions. Aircraft-based GHG measurements were the earliest atmospheric observations, pre-dating and inspiring the INFLUX project (Mays et al., 2009). INFLUX airborne GHG measurements were initiated in 2010 using the Purdue Airborne Laboratory for Atmospheric Research (ALAR), and include both continuous Cavity Ring-Down Spectrometer (CRDS) measurements of CO_2_, CH_4_ and H_2_O, and flask samples for subsequent laboratory analyses of 50 trace gases including ^14^CO_2_ (Turnbull et al., 2012). The CRDS was upgraded to include CO from the fall of 2014 through the summer of 2015. Cambaliza et al., (2014, 2015) and Heimburger et al., (2017) report average peak midday downwind enhancements of CO_2_ and CH_4_ of 5–10 ppm and 30–50 ppb, with considerable variability from day-to-day and across space, and discuss both instrument performance and the application of these observations to flux estimation (section 2.1.5) using the atmospheric mass balance approach.

Automobile-based GHG measurements have been conducted to identify point sources of methane (Cambaliza et al., 2015; Lamb et al., 2016). Automobiles were equipped with a CRDS (Picarro^1^) instrument and in some instances (Lamb et al., 2016) these were combined with SF_6_ tracers, measured with a custom-built continuous analyzer (Benner and Lamb, 1985; Flaherty et al., 2007), to infer point-source emissions.

**Figure 1:**
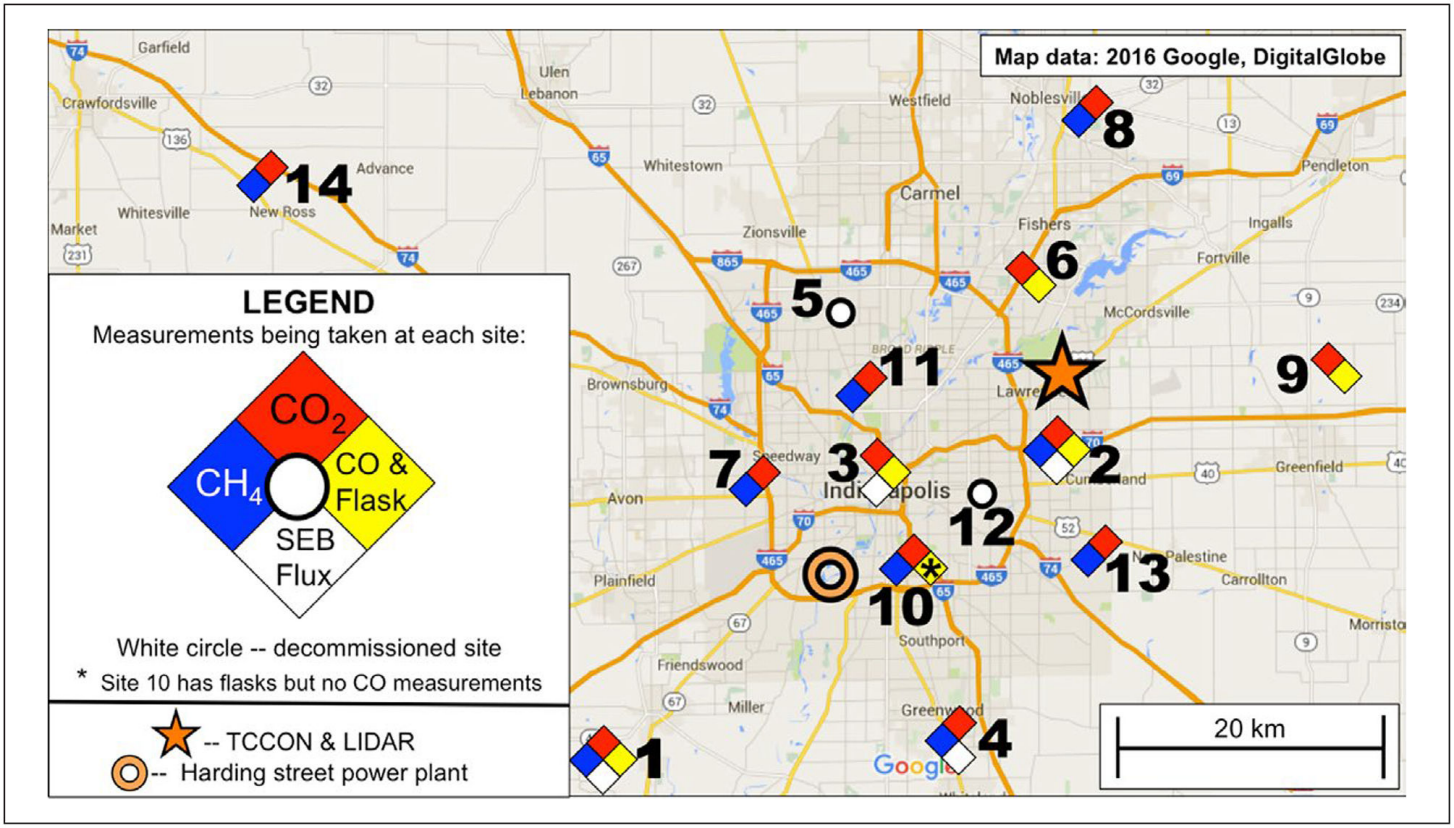
INFLUX GHG observational network. Map of the long-term observational network deployed for INFLUX including tower-based GHG and trace gas measurements, eddy covariance flux measurements, and ground-based remote sensing. Bold numers indicate the tower sites, and the colored diamonds indicate the measurements at each tower site. SEB flux refers to Surface Energy Balance flux measurements. Background imagery from Google, Inc. DOI: https://doi.org/10.1525/elementa.147.f1

Tower-based, continuous CRDS GHG measurements, mounted on existing communications towers, have been deployed at 12 long-term tower sites (Figure 1) across the Indianapolis region, with one short-term site (tower 12) that has since been decommissioned, and one long-term site (tower 5) that is being replaced with an additional background site (tower 14). Not all sites have been operational for the entire study. Richardson et al. (personal communication) outlines the tower instrumentation, the network evolution over time, the calibration methods, traceable to World Meteorological Organization (WMO) primary standards, and performance characteristics of these measurements. The continuous tower-based measurements demonstrate compatibility (defined as the difference between two measurements) of 0.18 ppm for CO_2_ and and 1.0 ppb for CH_4_ (Richardson et al., 2017). All towers include continuous CO_2_ mole fraction measurements, and a subset include continuous CH_4_.

The tower network continuously measures GHG mole fractions across the city, includes background mole fraction measurements from all wind directions and resolves GHG spatial patterns. This network provides the highest density of highly-calibrated GHG sensors in any environment, urban or otherwise, to date. The tower sampling heights were chosen to be as high as possible, with a goal of sampling uniformly at 100 m or more above ground level (AGL), since daytime vertical gradients are minimized at these altitudes (Bakwin et al, 1998; Wang et al, 2007). Limitations on the density and availability of existing towers, however, result in three towers with a maximum sampling height of 40 m AGL. Four towers include vertical profile measurements specifically intended to test the sensitivity of our regional GHG measurements to sampling altitude (Miles et al., 2017a). Analyses to date focus on mole fraction measurements in the well-mixed, daytime atmospheric boundary layer (ABL). Since the nocturnal boundary layer is highly stratified in the vertical and difficult to simulate, model-data comparisons of nocturnal observations would likely be dominated by uncertainty in atmospheric transport, not by GHG emissions. Miles et al. (2017a) show that the spatial-maximum, temporally-averaged urban CO_2_ and CH_4_ enhancements relative to the background tower 1 at midday in the dormant season are 2.9 ppm and 21 ppb when averaging all wind directions, and that this enhancement approximately doubles when sub-sampled for conditions when tower 1 is upwind. Miles et al. (2017a) also show that the enhancement varies considerably across the urban domain.

A subset of the towers (Figure 1) include flask sampling of GHGs (Turnbull et al., 2012). These data provide a second link, in addition to calibration gases, between our continuous, urban, CRDS GHG measurement network and WMO primary standards (Richardson et al., personal communication). Flask samples are collected on a roughly weekly basis in the afternoon, when winds blow from the southwest so that tower 1 (Figure 1) will serve as the upwind boundary condition, referred to as the “background” for the city. Samples are collected as one-hour integrated air samples to minimize short-term atmospheric variability that can confound interpretation. Flask samples have also been collected throughout the diurnal cycle on a few selected days.

#### 2.1.2 Additional trace gas measurements

Measurements of trace gases other than CO_2_ and CH_4_ enable source attribution among the multiple GHG sources that are collocated in the urban environment. The Purdue ALAR has recently been enhanced to include continuous measurement of CO for this purpose. Five of the tower sites (towers 1, 2, 3, 6, and 9; Figure 1) include continuous CO measurements. CO is a moderately good tracer of the production of CO_2_ from combustion (Turnbull et al., 2006, 2011; Levin and Karstens, 2007), though it is complicated by source-dependent variability in the CO:CO_2_ emission ratio, and by biosphere-atmosphere exchange of CO_2_ and CO precursors (Vogel et al., 2010; Turnbull et al., 2015). Richardson et al., (2017) document the calibration and performance characteristics of the CO measurements (compatibility of 6 ppb), and both Miles et al (2017a) and Heimburger et al (2017) report time-averaged, spatial-maximum midday urban CO enhancements of roughly 30 ppb, again with considerable day-to-day and spatial variation.

Many trace gases that may be useful in source attribution, including ^14^CO_2_, the best tracer of fossil fuel CO_2_ sources (Levin et al., 2003; Turnbull et al., 2006), at present cannot be measured continuously with useful accuracy and precision. An approach that enables a large number of different tracers to be analyzed is flask sampling. All five towers that include continuous CO measurements (sites 1, 2, 3, 6, and 9), one additional tower (site 10), and the ALAR have been equipped with a NOAA Global Monitoring Division (GMD) flask sampling system (Turnbull et al., 2012; Karion et al., 2013; Sweeney et al., 2015; Cambaliza et al., 2015). The tower-based samplers are activated remotely, and the aircraft system is operated manually. Samples are collected upwind and downwind of urban GHG sources in an effort to aid attribution, on a weekly basis as noted previously. The sampling frequency is low; this is a compromise dictated by the cost of flask collection and analysis. Turnbull et al. (2015) presented first analyses of these observations, demonstrating the need for a local upwind measurement of ^14^CO_2_, relatively little influence of biogenic CO_2_ on the urban enhancement during the dormant season, and a strong impact of biogenic CO_2_ on urban enhancements in the summer. Turnbull et al. (2015) also demonstrated that EPA inventories overestimated CO emissions by more than a factor of two, most likely due to an overestimate of the traffic emissions.

Automobile-based surveys including a Picarro CRDS measuring CO_2_, CO, CH_4_ and H_2_O, and a flask sampling system were also conducted and used to constrain traffic-specific multi-species and CO isotope emission ratios for Indianapolis (Vimont et al., 2017). These measurements were conducted while driving on major highways around Indianapolis and at busy intersections for two days in March 2015. The results support the urban traffic CO emission rate determined independently from tower measurements (Turnbull et al., 2015) and show that the Indianapolis traffic CO stable isotope values are significantly different from those of European cities, likely due to a small number of vehicles with uncatalysed exhaust (Vimont et al., 2017).

#### 2.1.3 Meteorological observations

Meteorological measurements in the region improve the accuracy and precision of the inference of GHG emissions from atmospheric GHG observations. We are using both operational and project-specific observations to evaluate and improve the atmospheric re-analyses used to infer GHG emissions from atmospheric GHG mole fractions.

**Figure 2:**
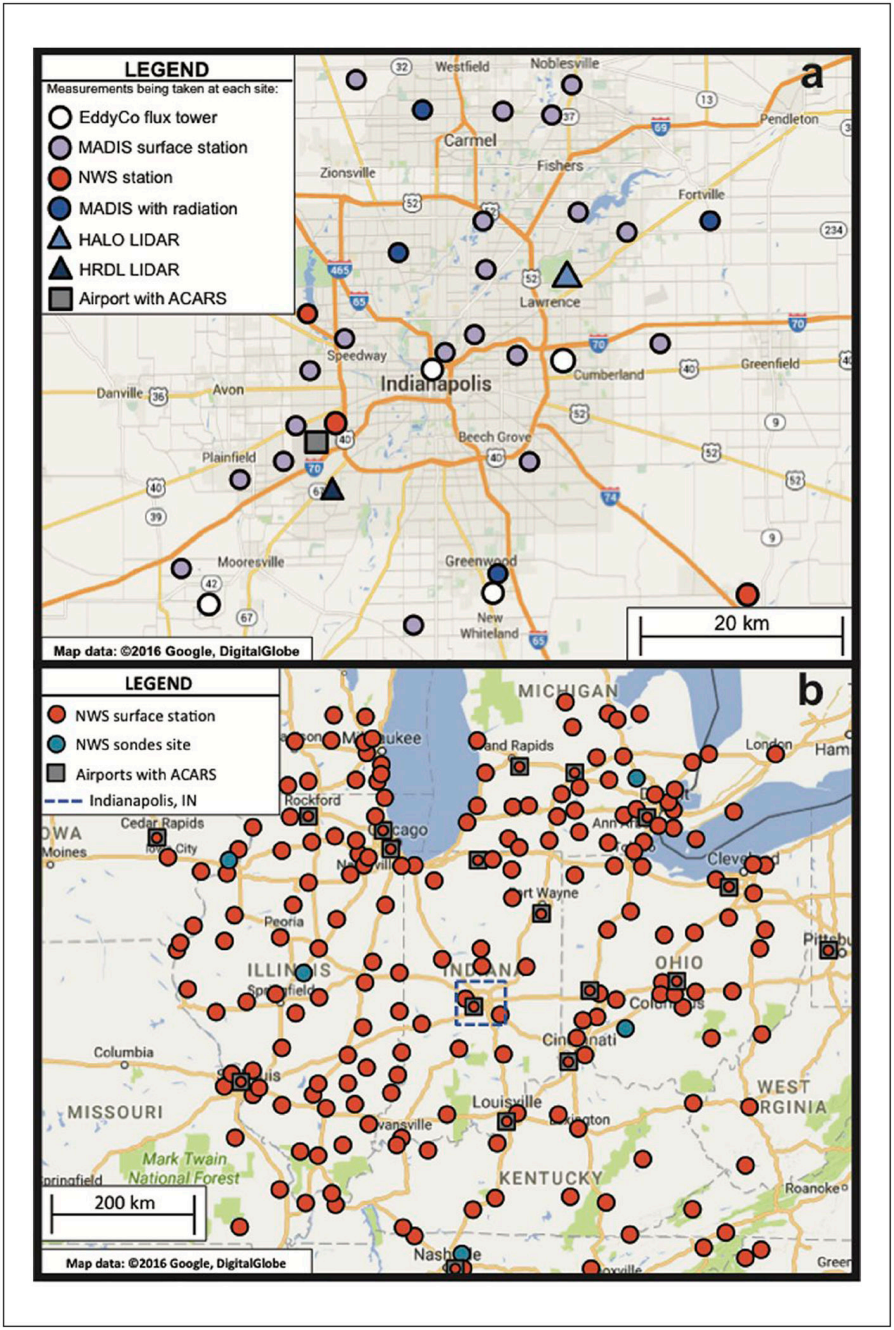
INFLUX meteorological observational network. Meteorological observational network supporting INFLUX within the Weather Research and Forecast model (WRF) **(a)** inner, 1 km resolution domain and **(b)** outermost, 9 km resolution domain. The inner domain is shown by the box outline in the center of (b). The MADIS surface stations are run by a number of organizations including the Indiana Department of Transportation, the Indiana Department of Environmental Management, and other private & public entities that contribute to the Citizen Weather Observer Program. Note that the maps do not correspond precisely to the model domains. DOI: https://doi.org/10.1525/elementa.147.f2

Existing observational networks, brought together in NOAA’s Meteorological Assimilation Data Ingest System (MADIS, 2017; Sarmiento et al., 2017a), provide considerable resources to describe the state of the atmospheric boundary layer in the region. A network of 24 surface stations operated by the National Weather Service, the Indiana Department of Transportation, the Indiana Department of Environmental Management, and other private & public entities that contribute to the Citizen Weather Observer Program (Figure 2a) provides surface layer wind, temperature and relative humidity observations. As a result of quality control tests (Steven Levine, National Centers for Environmental Prediction, personal communication), only a subset of measurements is used at some of the stations (Deng et al., 2017). Five stations supply incoming solar radiation measurements. Instrumented commercial aircraft participating in the Aircraft Communications Addressing and Reporting System (ACARS) program (Moninger et al., 2003, Anderson, 2010) provide intermittent but moderately frequent soundings of winds and temperature via flights in and out of Indianapolis International Airport. Regional National Weather Service surface and upper-air observations that meet WMO measurement standards are used to quantify atmospheric conditions in the domain surrounding the city (Figure 2b).

Surface energy balance and momentum flux measurements describe the interactions between the land surface and the regional ABL. Accurate simulation of the land surface is very important for accurate simulation of ABL winds and mixing depth. Four of the communications towers used for continuous atmospheric GHG measurements, towers 1, 2, 3 and 4 (Figures 1, 2a), also host eddy-covariance flux measurements of sensible and latent heat flux, momentum flux, and CO_2_ flux (Sarmiento et al., 2017a). These sites span the rural to urban gradient found in the region. Two of these towers (towers 1 and 3) also include measurements of hemispherically-integrated, upwelling and downwelling, solar and terrestrial radiation, another important element of the surface energy balance. The four towers were selected to represent rural settings (tower 1), dense urban settings (tower 3) and sites with intermediate levels of commercial (tower 2) and suburban (tower 4) development. The AmeriFlux network (Department of Energy, Lawrence Berkeley Laboratory, 2017) includes additional flux tower sites spanning the landscapes of the upper Midwest.

ABL properties including depth, mean wind, and turbulence are essential for interpretation of atmospheric GHG observations. These properties are continuously monitored by an autonomous Doppler lidar purchased from Halo Photonics^1^ (Pearson et al, 2009). Operating at a wavelength of 1.5 microns, the lidar is operated with a range resolution of either 38 m or 48 m and generates range-resolved estimates of radial velocity and backscatter signal intensity at a rate of 2 Hz. The precision of the radial velocity measurements is estimated to be better than 10 cm s^–1^ at high signal levels (Pearson et al, 2009). Every 20 minutes the lidar repeats a scan sequence to measure profiles of vertical velocity variance, boundary layer winds, and aerosol backscattered signal, which are used to estimate the mixed layer depth, ABL turbulent kinetic energy, and mean horizontal winds. The scan sequence includes a series of conical (Plan Position Indicator – PPI) scans at different elevation angles, vertical (Range Height Indicator – RHI) scans at two orthogonal azimuths, and an extended period during which the lidar stares vertically. The lidar is located on the east-central (predominantly downwind) side of the city (Figure 2a). Originally installed in April of 2013, the instrument was upgraded in early 2016 to improve its sensitivity for providing sustained coverage of ABL properties under low aerosol conditions. The scanning procedure was modified in 2016 to take advantage of the higher power and faster data rate, and now employs continuous scanning instead of stop and stare mode.

Research aircraft data, both multivariate and spatially extensive, can be used to document the meteorological state of the regional atmosphere. The Purdue aircraft, ALAR, provides infrequent but detailed, high-quality meteorological observations that can be used both for flux inference, and to evaluate atmospheric re-analyses. ALAR flights are primarily designed to measure GHG mole fractions and atmospheric properties (winds, temperature, pressure, and relative humidity) upwind and downwind of the city with high frequency, accuracy and precision (Garman et al., 2006, 2008). Some flights, designed specifically to evaluate our atmospheric modeling system, have followed a grid pattern across the urban domain to document a snapshot of the state of the ABL and lower free troposphere around the city.

#### 2.1.4 Atmospheric modeling

Inferring urban GHG emissions from atmospheric mole fraction observations requires knowledge of atmospheric transport. The atmospheric mass balance approach (Cambaliza et al., 2014, 2015; Heimburger et al., 2017) utilizes direct measurements of ABL winds and depth from the airborne platform to approximate atmospheric transport across the city. Another approach, which to date has been applied to interpretation of our tower-based GHG observations (Lauvaux et al., 2016; Miles et al., 2017; Deng et al., 2017), is to use a numerical weather prediction model, informed by atmospheric observations, to create a historical reanalysis of the state of the atmosphere. Large-scale atmospheric reanalysis products (e.g. Kalnay et al, 1996) have found broad utility as an approach to interpolate limited atmospheric observational networks across space and time. INFLUX is creating regional-scale, high-resolution reanalyses that include GHG sources, sinks and transport (Lauvaux et al., 2016; Deng et al., 2017).

We have adapted the Weather Research and Forecast model with atmospheric chemistry (WRF-Chem; Skamarock et al., 2008; Grell et al. 2005) to simulate the transport of GHGs. WRF-Chem can be formulated with many different domains, resolutions and parameterizations and, in historical mode, many different meteorological data sources can be used for assimilation to create a reanalysis product. The accuracy and precision of our inference of urban GHG fluxes depends on the accuracy and precision of these atmospheric reanalyses. We have taken two approaches to quantifying and mimizing atmospheric transport model error. One approach is assimilation of atmospheric observations. Data assimilation combines the strength of direct, local measurements of the atmospheric state with the representation of atmospheric governing equations contained in a numerical weather model. Deng et al., (2017) investigate the impact of assimilating INFLUX atmospheric observations on the regional atmospheric reanalysis product. Meteorological observations assimilated include all available WMO surface and upper-air winds, temperature and moisture fields, Doppler lidar winds, and ACARS winds and temperature. The assimilation strategy follows Rogers et al., (2013) and excludes assimilation of temperature and moisture within the model-simulated ABL. Deng et al. (2017) demonstrate large reductions in random error and modest reductions in biases in simulated ABL winds (speed and direction) by assimilating the INFLUX Doppler lidar data.

Another approach we are pursuing to reduce atmospheric transport model error is testing and ultimately improving model structure and input data. Sarmiento et al., (2017a) investigate the impact of updated land surface data, as well as land surface and ABL parameterizations on the accuracy and precision of the meteorological reanalysis. They find that model bias varies with season, that model performance outside the urban domain dominates systematic errors in ABL depth, and ABL wind speed and direction within the city, and that the ABL parameterization has more influence than both the land surface model and land surface data on model simulation of these ABL properties. Sarmiento et al., (2017a) demonstrate strong improvements in simulations of the surface energy balance via an improved land cover map, but the impacts on urban ABL winds and ABL depths are modest.

**Figure 3:**
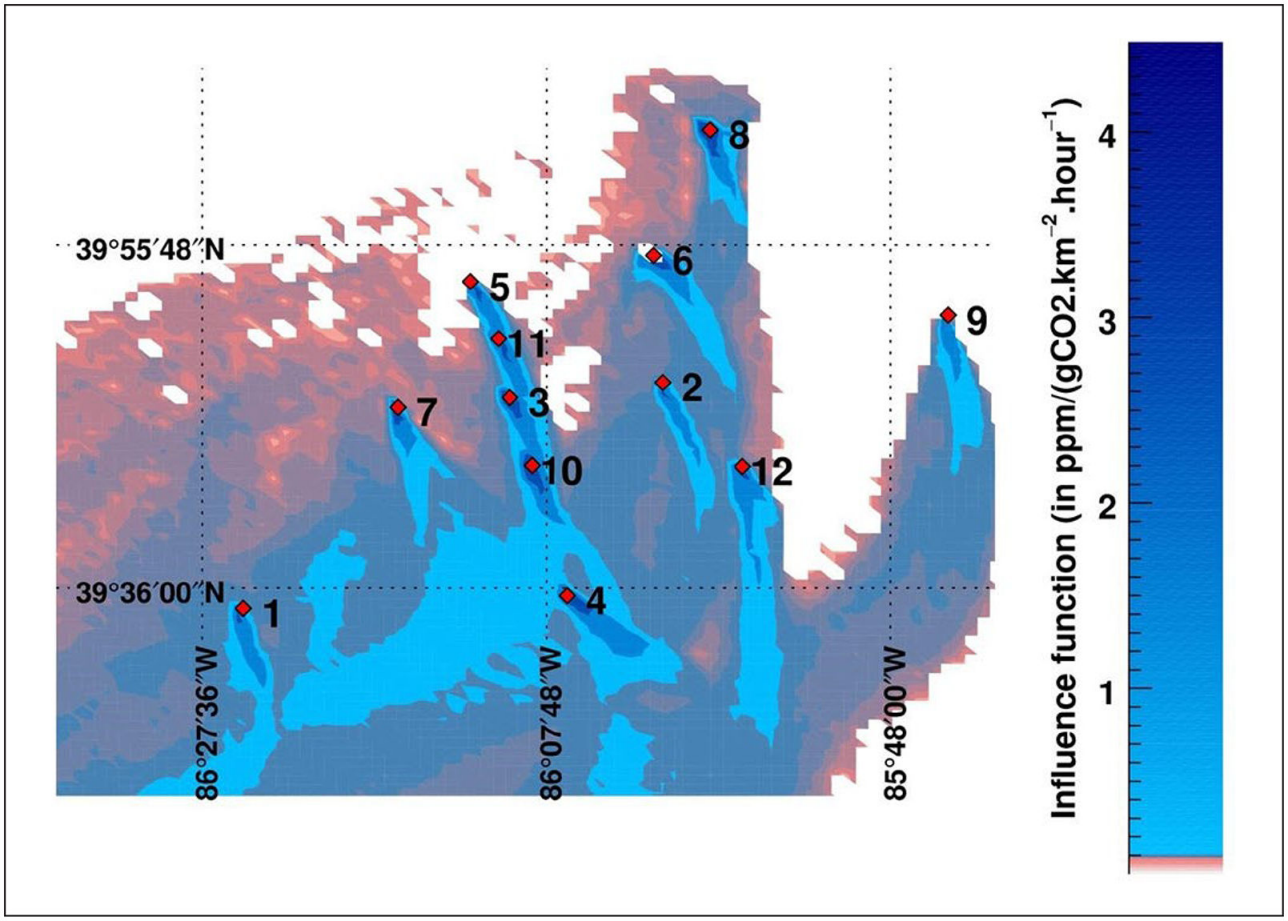
Tower influence function example. Total surface influence over a 12-hour window for observations from all towers collected over one hour beginning on 2 October, 2012 at 16 LST (22 UT). Numbers refer to the tower numbers, and the red diamonds give the locations of the towers. DOI: https://doi.org/10.1525/elementa.147.f3

INFLUX is also utilizing the Lagrangian Particle Dispersion Model (LPDM, Uliasz, 1994) to associate atmospheric GHG observations to regions in space and time whose GHG emissions influence the GHG mole fractions at those observation points. LDPM utilizes the wind and turbulence fields produced by WRF-Chem to run backwards-in-time trajectories from observations points, mapping out the surface areas that contribute to any given atmospheric GHG observation. Particles are “released” from the observation points (e.g. tower sites) and times, and tracked backwards in time. Times and places where the particles are within the atmospheric surface layer in this simulation are recorded, forming a function showing when the observations would be influenced by the surface (influence function). Convolution of the influence function with an estimate of surface fluxes yields the contribution of surface fluxes over the defined time period and within the chosen domain to the GHG mole fraction at the observation point. More details are given in Lauvaux et al., (2016) and Gaudet et al., (personal communication) and citations therein. Figure 3 shows an example of the calculated 12-hour influence functions for observations collected at 16 LST on 2 October, 2012, from the GHG observational towers, illustrating the capacity to quantify fluxes from different locations as wind speed and directions shift over time.

#### 2.1.5 Atmospheric flux inference methods

Mass balance is one approach utilized in INFLUX to infer GHG emissions. Urban GHG emissions are solved for analytically from an approximation of the ABL GHG conservation equation (Cambaliza et al., 2014, 2015). The net mass flow of incremental GHG mole fractions above a measured background are integrated across an imaginary plane perpendicular to the wind direction, and across all elements of the outflow plume. This relies on precise CRDS mole fraction measurements inside and out of the plume, as well as accurate wind speed measurements which are made using a Best Air Turbulence probe (Garman et al., 2006, 2008). Cambaliza et al., (2014, 2015) and Heimburger et al., (2017) report on uncertainties in the aircraft mass balance method for whole city emission determinations, as well as some source sector attribution and quantification. They show typical flight-by-flight uncertainties in urban emissions of roughly 30–40%. Heimburger et al (2017) investigate improvements in the aircraft mass balance method precision, via averaging, and find that CO_2_ and CO emissions rate uncertainty appears to be steady over a period of a few weeks, thus random errors can be reduced by repeated flights. Urban CH_4_ emissions, however, appear to be highly variable on the same time frame.

Another approach being utilized in INFLUX is a Bayesian matrix inversion that incorporates atmospheric GHG mole fraction measurements, the atmospheric transport reanalysis products, the LPDM, and a prior estimate of urban GHG emissions (Lauvaux, et al., 2016; Deng et al., 2017). At present we assume that the GHG boundary conditions for the city are represented by an upwind tower (Lauvaux et al., 2016; Miles et al., 2017a), and solve for GHG enhancements relative to this background measurement. This method also requires quantitative estimates of the uncertainties in the prior fluxes, atmospheric observations, and atmospheric transport reanalyses (Lauvaux et al, 2009, 2012a, 2016; Deng et al., 2017; Wu et al., personal communication).

We also measure local-scale (order 1 km^2^ footprint) CO_2_ fluxes directly at four towers (sites 1, 2, 3 and 4; Figure 1) using the eddy covariance technique (Sarmiento et al., 2017a). These measurements are intended to evaluate and/or be integrated into bottom-up flux estimates, and to evaluate the temporal dependence of inverse flux estimates.

#### 2.1.6 Bottom-up data products

Anthropogenic GHG emissions can also be estimated using a variety of economic, demographic, regulatory and land surface observations. INFLUX has primarily employed the Hestia data product (Gurney et al., 2012) for anthropogenic CO_2_ emissions estimates from Indianapolis (Marion County) and the surrounding eight counties. Hestia was developed using Indianapolis as a test case, and is designed to provide a high time (hourly) and space (down to the building level) resolution emissions estimate from multiple data sources. Hestia breaks down emissions according to different economic sectors (e.g. transportation, utilities, and residential, commercial and industrial), fuel, and combustion type (e.g. boilers, turbines, engines). INFLUX has also employed a night-lights based emissions estimate, the Open-source Data Inventory for Anthropogenic CO_2_ (ODIAC, Oda and Maksyutov, 2011) as an alternative to Hestia. ODIAC has less temporal and spatial resolution than Hestia and is not customized to Indianapolis, but is a global product, linking fossil fuel consumption data with satellite-based observations of light at night. Assessing products like ODIAC (or FFDAS, Asefi-Najafabady et al., 2014) that are not customized to an individual city helps to determine how well INFLUX methodology can be used in an urban region where a specialized inventory is not available.

INFLUX has not yet fully implemented a biological CO_2_ emissions inventory or process model. Many models exist for estimating CO_2_ fluxes from terrestrial ecosystems, but adaptation of these models to urban environments is a relatively recent endeavor (Kaye et al, 2006; Briber et al., 2015). Experimentation has been initiated with a very simple model, the Vegetation Photosynthesis and Respiration Model (VPRM, Mahadevan, et al., 2008; Hilton et al., 2013, 2014). Wu et al., (personal communication) use this model to explore the degree to which biological CO_2_ fluxes can mask anthropogenic GHG emissions in Indianapolis.

A custom CH_4_ emissions inventory was developed for INFLUX (Lamb et al., 2016) using a combination of local point flux measurements and traditional inventory-based activity data and emissions factors. This inventory separately quantified landfill, wastewater, and natural gas infrastructure, and all known, significant point sources. It included emission measurements from components of the natural gas infrastructure using a high-flow sampling method and plume dispersion measurements (Lamb et al., 2016).

### 2.2 Syntheses

INFLUX’s planned (but not necessarily inclusive) methodological elements have been implemented. Here we describe the plans for synthesis, and note progress towards the overall scientific objectives of high precision, accuracy and resolution urban GHG emissions estimates.

INFLUX is attempting to evaluate flux estimates by comparing our multiple flux methods, while also emphasizing uncertainty quantification within each method. Discrepancies, especially outside the bounds of quantified uncertainty, call for further investigation, including examination of the uncertainty bounds and the methods used to determine them. Agreement, but with unacceptably large uncertainties, calls for attempts to improve accuracy and precision. Success in achieving our objective of 10% accuracy and precision can be demonstrated both by uncertainty quantification within flux estimate methods, and via demonstration of consistent results across methods. The primary independent data we bring to bear are atmospheric GHG mole fraction measurements, both airborne and tower-based, and the economic, demographic and activity data used in bottom-up/socio-economic emissions inventory products.

Atmospheric flux inference methods detect all GHG fluxes into the atmosphere, and are influenced by relatively large source regions. As a result, spatial and temporal trends in atmospheric GHGs provide a powerful constraint on total GHG emissions. Aircraft and tower observations are complementary, as aircraft are excellent for covering a large area in a short period of time but by nature are not continuous, while tower-based sensors are excellent for continuous measurements, but are limited in spatial coverage. Limitations of atmospheric flux inference methods include limited source attribution, spatial resolution, and atmospheric transport accuracy and precision, and challenges quantifying the atmospheric background.

Spatial detail, and sector-specific input data are strengths of inventory products. Bottom-up GHG emission data products can be built upon detailed information concerning the magnitude, spatial distribution, and mechanistic details of sources. Challenges for inventory products include self-reported data of uncertain quality, methods that estimate emissions based on indirect approaches such as a mix of activity data and emission factors (Ogle et al., 2010; Cooley et al., 2013), difficulty updating estimates over time, and the potential of missing sources or sinks entirely depending upon the algorithms employed. New efforts to quantify emissions from the bottom-up have improved upon the initial, regulatory-driven efforts by using a larger mix of data sources, many of which overlap, generating greater reliability (Gurney et al., 2012; Gurney et al., 2017).

These three sources of insight into urban GHG emissions, airborne- and tower-based GHG measurements and inventory data products, are complementary, and largely independent. We plan to explore means of merging them into a single approach that meets the INFLUX research objectives. This strategy is similar to that employed by the North American Carbon Program (NACP) Midcontinent Intensive (MCI) regional study (Ogle et al., 2006), which resulted in a successful demonstration of independent quantification of CO_2_ fluxes from an agricultural region of the upper Midwest using both atmospheric (Miles et al., 2012; Lauvaux et al, 2012a) and inventory (Ogle et al, 2010; West et al, 2011) methods. Good agreement between these approaches (Schuh et al., 2013) was obtained. Ogle et al., (2015) outlined recommendations, as yet unrealized, for merging these approaches into a single, synthetic approach for regional flux determination.

#### 2.2.1 Whole-city emissions

INFLUX has demonstrated the ability to estimate whole-city GHG emissions using inventory (Gurney et al., 2012; Lamb et al, 2016), airborne (Cambaliza et al., 2014, 2015; Heimburger et al, 2017) and tower-based measurements (Lauvaux et al., 2016; Miles et al., 2017a), and has initiated cross-method comparisons (Lamb et al., 2016; Lauvaux et al., 2016). For CO_2_, an inventory assessment of fossil-fuel CO_2_ emissions for 2002 was documented by Gurney et al., (2012) and updated for 2011–2014, aircraft mass balance estimates have been published for 23 different days (Mays et al., 2009; Cambaliza et al., 2014, 2015; Heimburger et al, 2017), and a tower-based atmospheric inversion was used to estimate emissions from 8 months of the biologically dormant season (September, 2012–April, 2013, Lauvaux et al, 2016). A synthesis of published results is shown in Table 2. The current tower-based inverse CO_2_ emissions estimate is roughly 20% higher than the inventory-based anthropogenic CO_2_ estimate, and the difference appears to be statistically significant (Lauvaux et al., 2016). The uncertainties in tower-based CO_2_ emissions are estimated to be 10–15% over a time scale of eight months in the biologically dormant season (Lauvaux et al, 2016). The atmospheric inverse flux estimates are relatively insensitive to the choice of prior flux estimate, but that conclusion depends on the uncertainty in the prior fluxes, and the prior flux uncertainty structure is not known (Lauvaux et al., 2016).

Aircraft mass-balance flights have achieved similar precision for CO_2_ (approximately 15%) for a nine-flight average from a three-week campaign in the dormant season (Heimburger et al., 2017). The average of dormant season flux estimates, however, diverges from both inventory and tower-based inverse flux estimates (Table 2). A number of issues might explain the differences in fluxes in Table 2. The aircraft measurements do not encompass the same spatial and temporal domain as the tower inversion and inventory product, and the atmospheric methods will include biogenic CO_2_ fluxes while the inventory product does not. It is also important to note that the measurement uncertainties are more difficult to quantify than the urban emissions themselves. This INFLUX Special Feature contains considerable work aimed at further exploring and reducing these sources of uncertainty. Understanding and reconciling these differences is central to our experimental design and ongoing research efforts.

All three approaches have also been applied to the estimation of whole-city CH_4_ emissions (Table 2). Cambaliza et al. (2014, 2015) and Heimburger et al. (2017) have employed aircraft mass-balance methods, and Lamb et al., (2016) synthesized aircraft mass balance, inventory, and tower-based inverse flux estimates. Methane emissions estimates appear more variable with time, subject to greater methodological uncertainty, or both, as divergence within (Heimburger et al., 2017) and among (Lamb et al., 2016) methods has been found to be 50% or more of the mean emissions. Heimburger et al. (2017) concluded that the emissions themselves are likely more variable for CH_4_ than for CO_2_. Lamb et al. (2016) showed a significant discrepancy between tower-based emissions and aircraft-based fluxes, and a smaller discrepancy between aircraft and inventory flux estimates. While the aircraft and inventory results were within their respective uncertainty bounds, the tower-based results showed considerably larger emissions from the city. The tower-based emissions estimate in Lamb et al. (2016) was based on a relatively small number of towers; the number of towers measuring methane was expanded from five in April, 2013 to nine by November, 2014. Work to refine our quantification of CH_4_ emissions from Indianapolis continues.

#### 2.2.2 Spatially resolved emissions

Aircraft mass balance sampling was not intended for spatially resolved emissions estimates from Indianapolis, though a number of strong point sources have been quantified using this approach (Cambaliza et al., 2015). The relatively dense tower network (Figure 1) and influence functions that move with changing winds (Figure 3) provide some degree of spatial resolution in urban emissions. Inventory-based assessments provide very high spatial resolution, linked to the locations of built structures in the urban environment. At present, uncertainty in the spatial error structures in our existing inventory emissions estimates has limited our confidence in atmospheric inverse estimates of GHG emissions at any resolution finer than the entire city (Lauvaux et al., 2016; Wu et al., personal communication).

#### 2.2.3 Sectoral resolution

Both spatially-resolved atmospheric inverse flux estimates and atmospheric tracer measurements, especially CO and ^14^CO_2_, are intended to enable identification of the sources of urban CO_2_ emissions, and to complement the detailed sectoral information available from bottom-up data products. The most obvious need is to distinguish biogenic and anthropogenic CO_2_ fluxes. Distinguishing among anthropogenic sources is also of interest. To date most INFLUX studies of CO_2_ emissions have focused on the dormant season, when biogenic CO_2_ fluxes are weak compared to summer months. Turnbull et al. (2015) showed that from November to April, total urban CO_2_ enhancement above our local background was a good, though slightly biased, proxy for CO_2_ from anthropogenic sources. Summer conditions are more challenging. Nathan et al. (2017) explore the utility of multi-species data measured by the flask sampling network for identifying anthropogenic CO_2_ source sectors and find that because the major emissions sectors are not spatially separated from each other in a city like Indianapolis it is difficult to identify the source of each emission.

INFLUX has relied primarily upon spatial information for distinguishing CH_4_ sources. Since CH_4_ emissions are dominated in the city by a small number of large sources (Cambaliza et al., 2015; Lamb et al., 2016), this has been a relatively successful approach. Lamb et al. (2016) explored the use of continuous ethane measurements for identifying methane sources, but INFLUX does not currently include continuous ethane measurements as part of its long-term tower network.

#### 2.2.4 Temporal trends

High fidelity, long-term monitoring of atmospheric GHG mole fractions and the state of the urban ABL are intended to provide the accuracy and precision in inverse GHG flux estimates needed to identify changes in GHG emissions over time. Atmospheric data have been demonstrated to be uniquely capable of identifying changes in urban emissions over time (Lauvaux et al., 2013). INFLUX has quantified uncertainties in urban emissions estimates, but has not yet explicitly examined trend detection. Multi-year flux estimates are a high priority for future research using the INFLUX observational array.

#### 2.2.5 Observational system tests

INFLUX is intended as a testbed for urban GHG emissions monitoring. This can be done both with data removal or data degradation experiments using the existing observational network, or by hosting additional experimental observations or analytic methods. Wu et al., (personal communication) show that both improvements in atmospheric transport modeling and improved knowledge of prior flux errors should substantially improve our inverse flux estimates, while degredation in atmospheric CO_2_ observations, especially the introduction of biased data, would significantly degrade the quality of CO_2_ flux inversions. Lauvaux et al. (2016) demonstrated that while the whole city CO_2_ emissions estimates are not highly sensitive to removal of some of the existing tower network, spatial emissions patterns are quite sensitive, very similar to the results found with the NACP MCI network (Lauvaux et al., 2012b). Deng et al. (2017) examine the sensitivity of transport and inverse flux estimates to local atmospheric meteorological data, and show, consistent with Wu et al., (personal communication), notable improvements in both atmospheric transport and in the quality of inverse CO_2_ flux estimates. Many more experiments can be envisioned to quantify our ability to determine urban GHG emissions as a function of investment in observational and modeling infrastructure.

## 3. Current challenges in determining urban GHG emissions

A number of challenges confront our effort to achieve INFLUX’s scientific objectives. These challenges are not unique to INFLUX. We present a brief review of the major issues.

### 3.1 Sector attribution and biogenic fluxes

Preliminary results from INFLUX suggest that distinguishing anthropogenic from biogenic CO_2_ fluxes, particularly in the summer when biological CO_2_ fluxes are large, will be challenging. Wu et al., (personal communication) show that accurate, continuous observations of CO_2_ of fossil origin, if technologically feasible, would enable segregation of biogenic urban CO_2_ fluxes from urban fossil fuel CO_2_ emissions and retain comparable accuracy in the fossil emissions estimates to those obtained in the dormant season.

The best current tracer of fossil fuel CO_2_ is ^14^CO_2_, which at present can only be measured with sufficient accuracy using flask samples (e.g. Turnbull et al., 2015). Turnbull et al. (2015) showed, however, that the enhancement in downwind total atmospheric CO_2_ increases significantly in the summer months. This may be caused by a summer increase in urban anthropogenic or biogenic CO_2_ emissions, but is most likely caused by biogenic fluxes upwind of the city. The ideal tracer for anthropogenic CO_2_, ^14^CO_2_, is difficult to measure so data density is poor, and the most obvious tracer that is relatively easier to measure, CO, may be contaminated by photochemical sources in the summer (Turnbull et al, 2015; Vimont et al., 2017). We can improve our ability to determine summer anthropogenic CO_2_ emissions by improving our understanding of the summer CO_2_ background, production of CO from oxidation of biogenic hydrocarbons, and urban biogenic CO_2_ fluxes.

Sectoral attribution from atmospheric measurements is also challenging. Individual, large sources such as landfills (CH_4_) and power plants (CO_2_) can be isolated spatially in aircraft measurements (Cambaliza et al., 2014, 2015) and by their distinctive trace gas signatures. For example, the power plant is distinguished by abundant CO_2_ but negligible CO emissions (Turnbull et al., 2015) Other sources appear to be “well mixed” across the urban landscape, and influence functions integrate across these sources. This mixing gives tower-based measurements little ability to distinguish among these sectoral emissions without a priori information about the trace gas profiles of each emission source (Nathan et al., 2017). New sampling strategies and more detailed information about the mixture of trace gases produced from each source sector need to be considered if we are going to be able to isolate individual source sectors within the urban environment.

Spatial resolution in flux estimation can provide information about sectoral emissions. The tower-based inversions can be compared to the inventory-based assessments, providing, at some spatial resolution, a cross-comparison of these methods (e.g. Ogle et al., 2015). Atmospheric CH_4_ data density has increased substantially, and may yield more sector-specific information about CH_4_ sources. An understanding of the spatial structures in prior flux uncertainties, however, is critical to proper interpretation of such a comparison (Lauvaux et al., 2016; Wu et al., personal communication).

### 3.2 Atmospheric sampling and modeling

Accurate and precise determination of background GHG mole fractions is essential for both airborne and tower-based urban GHG emissions estimates. This can be challenging due to spatial heterogeneity in the background. This is particularly challenging for CO_2_ in the growing season due to the combination of strong and spatially heterogeneous biological fluxes, and strong diel variations in ABL mixing (Turnbull et al., 2015). Our current approaches to background estimation (Cambaliza et al., 2014; Lauvaux et al., 2016; Miles et al., 2017a; Heimburger et al., 2017) are a significant source of uncertainty. Paths forward include installation of a second background site (tower 14), synthesis of tower and aircraft data to encompass temporal and spatial variability in background conditions, and simulation of the impacts of biogenic CO_2_ fluxes and regional CH_4_ emissions on atmospheric background conditions.

Interpretation of atmospheric GHG data is limited by our ability to simulate atmospheric transport. One category of problems arises from the fact that in many cases our GHG measurements are fairly close to the sources. Measurements close to strong point sources (Gaudet et al., personal communication), or close to the surface (Miles et al., 2017a) are influenced by near-field turbulent mixing which our current mesoscale atmospheric modeling system has limited capacity to simulate. Gaudet et al., (personal communication) explore the sensitivity of WRF’s parameterization of atmospheric dispersion by comparing the mesoscale model to dispersion theory and a turbulence-resolving implementation of the WRF model. They find that WRF overestimates vertical dispersion for sources within one or two eddy turnover times (tens of minutes of advection time, or 5–10 km in typical conditions) of the observation point (tower or aircraft). This bias is strongest close to the source. Improved representation of turbulent dispersion close to our observation points is needed to correct this bias.

A similar problem arises with aircraft mass balance when the flights are conducted closer to major sources than a few eddy turnover times. However, moving the measurements farther from the source regions reduces the atmospheric signal (Miles et al., 2017) and enhances the impacts of background uncertainty, and would reduce our ability to distinguish sources using spatial information. Near-field effects can be treated with turbulent dispersion theory, large-eddy simulations (Gaudet et al., personal communication), and micrometeorological observations (Wang et al., 2007). Combining this understanding with knowledge of the locations of strong point sources can quantify and minimize these potential biases.

Another challenge with interpretation of atmospheric GHG data arises from our limited knowledge of atmospheric transport at the spatial scales that are resolved by mesoscale atmospheric models. Imperfect parameterizations of atmospheric processes and sparse measurements of the atmospheric state and boundary conditions result in errors in atmospheric transport reanalyses (Deng et al., 2017) that impact the simulated atmospheric GHG fields (Díaz et al., 2014). Imperfect knowledge of atmospheric mixing, especially ABL depth, ABL wind speed and direction, and ABL turbulence create errors, both random and systematic, in our inference of GHG emissions from atmospheric data (Gerbig et al., 2008; Lauvaux and Davis, 2014; Deng et al., 2017). The complex urban surface creates additional challenges in simulating atmospheric transport (Sarmiento et al., 2017a). Meteorological data assimilation (Deng et al., 2017) and comparative evaluation of model physics ensembles (Sarmiento et al., 2017a) will continue to guide improvements in our mesoscale atmospheric modeling systems.

These challenges in simulating atmospheric transport are exacerbated by nighttime conditions when turbulent eddies are small, and the atmosphere can be stable and highly stratified in the vertical. At present we do not utilize nighttime data due to the expectation that errors in simulated atmospheric mixing would overwhelm information about GHG fluxes in interpretation of those data. The lack of nighttime atmospheric GHG constraints leaves only the inventory data to constrain nighttime emissions. Improved understanding of transport in the stable ABL could greatly expand our ability to constrain urban GHG emissions with atmospheric data, and is an important topic for future study.

### 3.3 Inventories and emissions modeling, and synthesis with atmospheric data

The scientific objectives of INFLUX demand significant advances in the development of urban emissions models and inventories. While inventory products such as Hestia achieve very high spatial resolution, the temporal resolution sought by INFLUX exceeds the current limits of inventory products. Urban biological modeling and carbon accounting is still in its infancy. Ultimately, these prior emissions estimates can be merged with atmospheric data to create an urban carbon balance that is constructed from multiple constraints. This assimilation requires careful quantification of the uncertainties in these inventory products and models, including both the magnitude of the uncertainty and the correlations of these uncertainties across space and time. Advances in quantification of the uncertainties in these bottom-up flux estimates should lead to significant advances in joint application of top-down and bottom-up methods, and resulting understanding of urban GHG emissions. Development of high-resolution inventories with methods that can be rapidly extended to other cities (Oda et al., 2017) will enable expansion of INFLUX methods to other urban settings.

### 3.4 Metrics for success

How close have we come to achieving INFLUX’s stated goals to quantify CO_2_ and CH_4_ emissions at 1 km^2^ resolution with a 10% or better accuracy and precision, to determine whole-city emissions with similar skill, and to achieve high (weekly or finer) temporal resolution at both spatial resolutions? This objective is within sight for whole-city, dormant season CO_2_ emissions. Both aircraft mass-balance (Heimburger et al., 2017) and tower-based inversion results (Lauvaux et al., 2016) document whole-city CO_2_ emissions estimates during the dormant season at roughly 15% uncertainty with a temporal resolution of weeks to months. These methods have not yet been shown to converge to within that level of uncertainty, and both differ more than 10% from the Hestia inventory (Gurney et al., 2012), but it should be noted that the estimates do not necessarily represent the same emissions. Gurney et al. (2017) investigate issues including urban biogenic CO_2_ fluxes that might explain the difference between CO_2_ inventory and inversion results. The aircraft mass balance estimates are limited in their temporal and spatial coverage. Achieving convergence among methods and overall uncertainty of 10% or less in whole-city, dormant season CO_2_ emissions appears to be a tractable near-term goal. This should enable independent verification of trends in urban emissions in Indianapolis or other cities where comparable observational systems are deployed.

Whole-city CH_4_ emissions, and CO_2_ emissions during the growing season present more challenges. Our CH_4_ estimates disagree more (Lamb et al., 2016) than our CO_2_ emissions estimates, and uncertainty within methods is greater (Lamb et al., 2016; Heimburger et al., 2017). It may be that temporal variability in emissions is greater for CH_4_ than for CO_2_, and that our methodological accuracy and precision in estimating emissions is similar, but this hypothesis requires additional investigation. We have not yet demonstrated quantification of CO_2_ emissions during the growing season, but progress is being made, and uncertainty quantification of our first estimates should be available shortly. High accuracy and precision emissions estimates at high spatial and/or sectoral resolution is likely the most challenging of our objectives, and will almost certainly require joint progress in both inventory and atmospheric methods, including careful uncertainty estimation in each approach.

Perhaps the greatest challenge for INFLUX is identification of the metrics that must be achieved for urban GHG emissions monitoring to be successful. Local (urban or regional) emissions measurements are not yet utilized in an operational fashion. The suite of methods we are applying in INFLUX are clearly complementary, and provide unparalleled insight into urban GHG emissions. It is not clear, however, what aspects of these methods will prove useful for operational application. It is likely that multiple metrics for success will exist depending on the application. Collaboration with potential stakeholders in the monitoring of urban GHG emissions will help to refine future research directions and the expectations that drive them.

## 4. Future initiatives

We intend to make INFLUX a testbed for development of urban GHG emissions monitoring technology. The observational and numerical infrastructure, and knowledge base that exists can facilitate testing of new approaches – either numerical or observational – to improving our understanding of the urban carbon cycle. Continued observations, ongoing evaluation of the essential elements of the observational network, and readily accessible documentation of existing data and numerical methods are necessary to creation of an effective testbed facility. The INFLUX Special Feature is one contribution to this effort.

## Figures and Tables

**Table 1:** Methodological components of the Indianapolis Flux Experiment (INFLUX). DOI: https://doi.org/10.1525/elementa.147.t1

Component	Measurements/Instruments/Models	Description/Purpose	Data/Status	Data archive/References
Aircraft mass balance flights	Airborne Laboratory for Atmosphere Research (ALAR); Meteorological variables,^1^ continuous CO_2_. CH_4_, H_2_O and CO, flask samples.^2^	Measure GHG mass balance across the city. Evaluate simulations of atmospheric GHGs.	54 mass balance flights, 10 grid flights; February, 2008 - December, 2016.	Shepson et al., 2017/Mays et al, 2009; Cambaliza et al., 2014, 2015; Heimburger et al, 2017.
			350 flasks from 48 flights/Ongoing.	
Automobile surveys	Continuous CO_2_, CH_4_ and CO, flask samples.^2^	Surveys to identify strong point sources. Point source estimation via dispersion models.	500 kilometers of road data, 2012–2014. Completed.	No public archive/Cambaliza etal, 2015; Lamb et al., 2016; Vimont et al., 2017.
		Evaluation of multi-species and isotope ratios from specific source sectors.	Source sector surveys, 2015./Completed.	
Tower-based GHG and atmospheric tracer network	Continuous CO_2_, CH_4_ and CO, flask samples.^2^	Quantify urban GHG enhancement. Tower-based inverse flux estimates.	2 to 12 tower sites, continuous operation. 1,600 flasks from 375 unique dates and 7 towers./December, 2010 - October, 2016.	Miles et al., 2017b/TurnbuIl et al., 2012, 2015 Richardson et al., 2017; Miles et al., 2017a.
			Ongoing.	
Meteorological measurements	Eddy covariance and radiative fluxes^3^	Atmospheric state measurements. Atmospheric transport model assimilation and evaluation.	4 towers with eddy covariance, 2 with radiative fluxes; 2013 to present./Ongoing.	Sarmiento et al., 2017b/Sanniento et al., 2017a.
	Doppler lidar^4^		One site, continuous, 2013 to present./Ongoing.	Brewer, 2017
	Research aircraft^1^		See aircraft mass balance flights, above.	See aircraft mass balance flights.
	Surface weather stations		20–24 sites, continuous,/Ongoing.	MADIS, 2017/Sarmiento et al., 2017a; Deng et al, 2017
	Commercial aircraft^5^		Indianapolis International airport, approx. 15 flights per day, continuous./Ongoing	MADIS, 2017/Moninger et al, 2003
Column carbon observations	Continuous column CO_2_, CH_4_, CO.	Comparison to tower GHG network; urban GHG flux estimation.	TCCON: August - December, 2012. Completed. EM27 network, May, 2016. Completed.	Oak Ridge National Laboratory, 2017
Atmospheric transport modeling	Weather Research and Forecast Model fVVRF) with Chemistry (Chem) and Large Eddy Simulation (LES) options.	Simulation of atmospheric transport of GHGs; meteorological data assimilation; turbulence-resolving simulations	Continuous nested simulation from September, 2012 - October, 2015.	Sarmiento et al. 2017c/Lauvaux et al., 2016; Deng et al, 2017; Sarmiento et al, 2017; Gaudet et al, personal communication.
			Physics ensemble simulation for a winter month (15 February- 20 March, 2013) and a summer month (15 June - 20 July 2013).	
			WRF-LES simulation of 28 September, 2013.	
Atmospheric inversion system	Lagrangian Particle Dispersion Mode! (L.PDM); Bayesian matrix inversion	Receptor - source attribution; Elux estimation integrating atmospheric transport, prior flux estimates, and atmospheric GHG observations	LPDM influence functions from September, 2012.-October, 2015.	No public archive./Lauvaux et al., 2016; Deng et al, 2017; Wu etal, personal com munication.
GHG flux estimates	Mesoscale atmospheric inversion	Whole city and spatially resolved GHG flux estimation	Tower-based flux estimates for CO_2_ and CH_4_; September, 2012. - April, 2013.	No public archive./Lauvaux et al., 2016; Lamb etal., 2016.
			Ongoing.	
	Atmospheric mass balance	Whole cityGHG flux estimation	68 flights including mass balance, grid and eddy covariance, 2009 – 2016./Ongoing.	Documented in publications./Mays et al., 2009; Cambaliza et al., 2014, 2015; Heimburger et al, 2017.
	Eddycovariance	Local area (~1 km^2^), continuous flux measurement	CO_2_ fluxes at 4 towers, 2013 - early 2016./Ongoing.	Sarmiento et al., 2017b.
	Plume inversion; Enclosures.	Point source GHG flux estimates	Survey in 2.013.	No public archive./Lamb et al., 2016.
Emissions inventories/data products	Activity data, fuel statistics, stack monitoring, model algorithm, emission factors	“Bottom-up” estimate of CO_2_, and CH_4_ fluxes from the city.	Anthropogenic CO_2_: 2002, 2010–2014.	No public archive./Gurney etal., 2012; Lamb et al., 2016.
			Ongoing.	
			Total urban CH_4_: Single assessment.	
			Completed.	

1 Three dimensional winds, temperature, and pressure at 50 Hz.

2 50 trace gases including CO_2_, CH_4_ and ^14^CO_2_. Complete documentation provided by Turnbull et al., (2012).

3 Terrestrial and solar, upwelling and downwelling hemispheric radiation, turbulent fluxes of virtual temperature, momentum, water vapor and CO2.

4 Mean horizontal winds, turbulent velocity along-beam, aerosol backscatter, ABL depth, turbulent kinetic energy.

5 Horizontal winds, temperature, pressure at 1 to 30 second resolution.

**Table 2:** Indianapolis whole-city CO_2_ and CH_4_ emission estimates published to date. Aircraft mass balance data are averages from the following flight days: 2008 (3/28, 4/2, 2/14, 2/15, 4/21, 11/28, 12/20); 2009 (1/7); 2011 (3/1, 4/29, 6/1, 6/30, 7/12); 2012 (11/8); 2014 (11/13, 11/14, 11/17, 11/19, 11/20, 11/21, 11/25, 12/1, 12/3). The three summer dates in 2011 are excluded from the CO_2_ aircraft mass balance results due to complications with background conditions in the summer. The confidence interval for the aircraft mass balance average is twice the standard error of the individual estimates. Aircraft mass balance flux estimates represent average emissions from a time window starting few hours before the midday to afternoon flight times. Tower inversion and inventory emissions represent best estimates averaged over the entire time periods noted in the table, including day and night. The area encompassed by the airborne mass balance estimates includes most of the city, but varies somewhat from flight to flight. The tower inversion and inventory estimates represent an 87 *×* 87 km^2^ region centered on the city. DOI: https://doi.org/10.1525/elementa.147.t2

	Aircraft mass balance	Tower inversion	Inventory product
Urban CO_2_ emissions (mol s^−1^)	14,000	22,600	18,200
Uncertainty (mol s^−1^)	3,300 (95% CI)	20,800 – 23,400 (25^th^-75^th^ percentile)	Not yet estimated
Time domain	2008–9, 2011–12, 2014.	Sept. 2012 - Apr. 2013.	Sept. 2012 - Apr. 2013
References	Cambaliza et al, 2014, 2015; Heimburger et al, 2017.	Lauvauxetal., 2016	Gurneyet al., 2012
Urban CH_4_ emissions (mol s^−1^)	103	160	57
Uncertainty (95% CI) (mol s^−1^)	27	147 – 174	30 – 107
Time, space domain	2008–9, 2011–12, 2014	Sept. 2012 - Apr. 2013	2013
References	Cambaliza et al., 2014, 2015; Heirnbureer et al, 2017.	Lamb et al., 2016	Lamb et al., 2016

## Footnotes

1 Certain commercial equipment, instruments, or materials are identified in this paper in order to specify the experimental procedure adequately. Such identification is not intended to imply recommendation or endorsement by the National Institute of Standards and Technology, nor is it intended to imply that the materials or equipment identified are necessarily the best available for the purpose.
